# The Critical Role of Membrane Cholesterol in *Salmonella*-Induced Autophagy in Intestinal Epithelial Cells

**DOI:** 10.3390/ijms150712558

**Published:** 2014-07-15

**Authors:** Fu-Chen Huang

**Keywords:** cholesterol, *Salmonella*, Intestine epithelia, autophagy

## Abstract

It was previously observed that plasma membrane cholesterol plays a critical role in the *Salmonella*-induced phosphatidylinositol 3-kinase-dependent (PI3K)-dependent anti-inflammatory response in intestinal epithelial cells (IECs). The PI3K/Akt pathway is associated with autophagy which has emerged as a critical mechanism of host defense against several intracellular bacterial pathogens. Plasma membrane contributes directly to the formation of early Atg16L1-positive autophagosome precursors. Therefore, this study aimed to investigate the role of plasma membrane cholesterol on the *Salmonella*-induced autophagy in IECs. By using methyl-beta-cyclodextrin (MBCD), it was demonstrated that disruption of membrane cholesterol by MBCD enhanced NOD2 and Atg16L1 proteins expression in membrane, and autophagic LC3II proteins expression and LC3 punctae in *Salmonella*-infected Caco-2 cells, which was counteracted by Atg16L1 siRNA. Nucleotide-binding oligomerization domain-containing protein 2 (NOD2) siRNA enhanced the *Salmonella*-induced activation of Akt in Caco-2 cells. However, inhibitors of Akt or extracellular signal-regulated kinases (ERK) had no significant effect on *Salmonella*-induced autophagy Beclin 1 or LC3 proteins expression. In conclusion, our study suggests that cholesterol accumulation in the plasma membrane at the entry site of *Salmonella* results in the formation of *Salmonella*-containing vacuole (SCV) and decreased autophagy. Our results offer mechanistic insights on the critical role of membrane cholesterol in the pathogenesis of *Salmonella* infection in intestinal epithelial cells and the therapeutic potential of its antagonists.

## 1. Introduction

Autophagy has emerged as a critical mechanism of host defense against several intracellular bacterial pathogens [1]. Plasma membrane contributes to autophagosome formation [2,3] as a control center for autophagy [2], leading to clearance of intracellular microorganisms. Recently, it has become increasingly evident that lipids and lipid-modifying enzymes play critical roles in the autophagy process, both as regulators of autophagy and as constituents of autophagic membranes [4]. Besides, plasma membrane cholesterol plays a crucial role on the *Salmonella*-induced PI3K/Akt-mediated anti-inflammatory responses in intestinal epithelia cells (IECs) [5], that may contribute to the establishment of *Salmonella* in the intestine [6]. Thus, events at the plasma membrane regulating biogenesis of autophagy are worth investigating.

Autophagy recognizes the population of *Salmonella* enterica serovar *typhimurium (S. typhimurium)* within damaged *Salmonella*-containing vacuoles (SCVs) early after infection in order to prevent bacterial escape [7,8]. PI3K/Akt recruited to SCV membrane may play a role in this autophagy pathway. Inhibition of Akt signaling [9] and enhanced ERK 1/2 activity [9,10] were associated with autophagy in human colon adenocarcinoma cells. On the other hand, two groups of investigators [11,12] have demonstrated that nucleotide-binding oligomerization domain-containing protein 2 (NOD2) is critical for the autophagic response to invasive bacteria because they recruit autophagy-related protein 16-like 1 (ATG16L1) to bacterial entry sites at the plasma membrane. The abnormalities in the handling of intracellular bacteria through autophagy might play a role in Crohn’s disease pathogenesis [13,14,15,16,17]. Therefore, it is reasonable to speculate that membrane cholesterol may play a critical role in the *Salmonella*-induced autophagy in IECs via the PI3K/Akt or NOD2-Atg16L1 pathways.

This study aimed to investigate the role of membrane cholesterol in *Salmonella*-induced autophagy in IECs. Although no literature has been documented on the effect, it would be an important issue to elucidate the role of this interaction on the molecular pathogenesis of *Salmonella* infection and Crohn’s disease.

## 2. Results and Discussion

### 2.1. Depletion of Membrane Cholesterol Induced the Activation of Autophagy Proteins Expression in Salmonella-Infected Intestinal Epithelial Cell (IEC)

**Figure 1:**
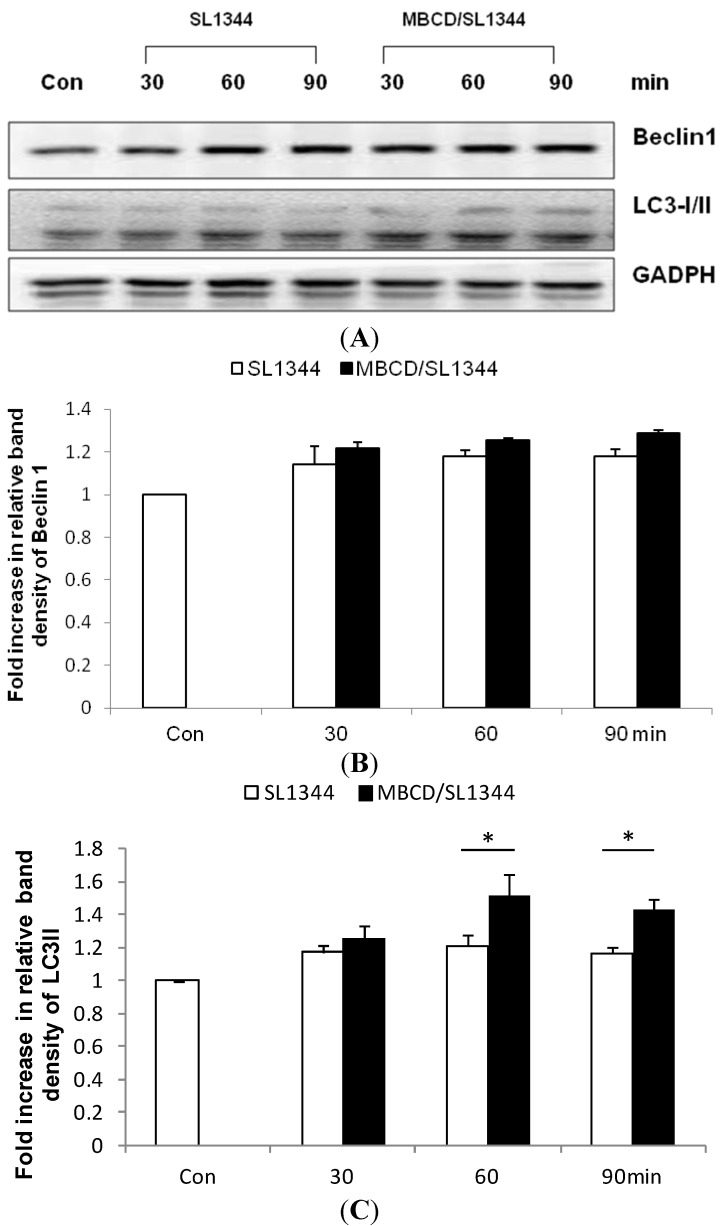
The role of membrane cholesterol on the autophagy proteins expression in *Salmonella*-infected Caco-2 cells. Caco-2 cells were uninfected (Con) or infected by wild-type *S*. *typhimurium* strain SL1344 at indicated times, in the presence or absence of methyl-beta-cyclodextrin (MBCD). Immunoblots were performed on whole cell lysates with an antibody to detect autophagy Beclin 1 and LC3II proteins expression, or GAPDH for normalization of proteins. Representative immunoblots (**A**) and densitometric quantification of immunoreactive bands (**B**,**C**) were shown; The relative band intensities of Beclin 1 (**B**) and LC3II (**C**) in untreated (white) and treated (black) Caco-2 cells were quantified as fold increases compared with the control cells. Each value represents the mean ± standard error of mean (SEM) of 3 independent experiments. An asterisk indicates a significant difference (*p* < 0.05).

In order to exploit if membrane cholesterol is involved in the *Salmonella*-induced autophagy in intestinal epithelial cells (IECs), Caco-2 cells were treated with methyl-beta-cyclodextrin (MBCD), and then infected by wild-type *S*. *typhimurium* strain SL1344. Immunoblots were performed on whole cell lysates with antibody to detect LC3II and Beclin 1 expression, two of the most important markers of autophagy. As shown in Figure 1, cholesterol depletion by MBCD enhanced autophagy markers LC3II but not Beclin 1 expression in *Salmonella*-infected Caco-2 cells.

### 2.2. The Involvement of ERK and Akt in Beclin 1 or LC3II Proteins Expression in Salmonella-Infected IEC

**Figure 2:**
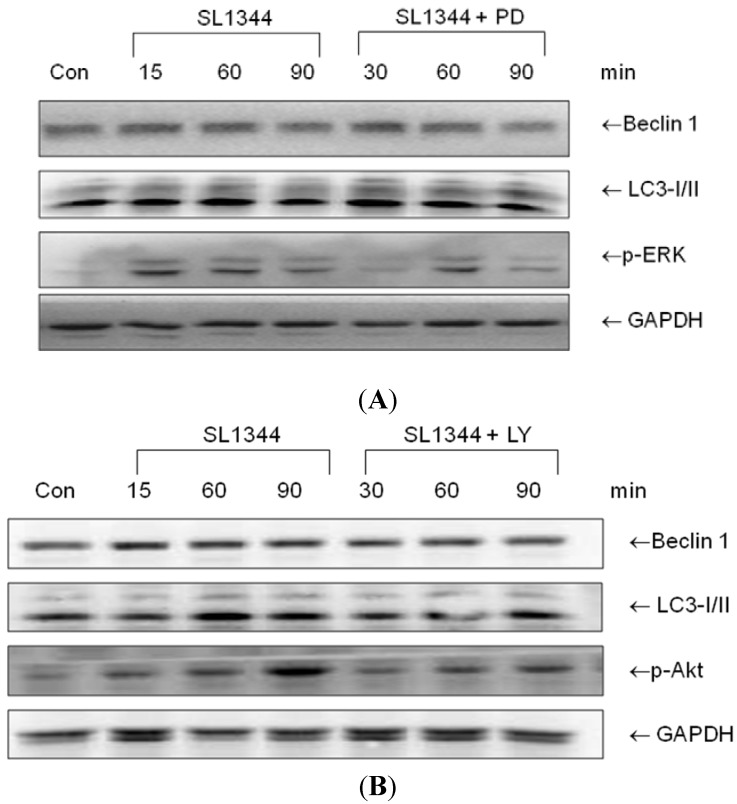
The role of ERK or Akt on the Beclin 1 and LC3II proteins expression in *Salmonella*-infected Caco-2 cells. Caco-2 cells were uninfected (Con) or infected by wild-type *S*. *typhimurium* strain SL1344 for indicated times, in the presence or absence of PD98059 (PD) or LY294002 (LY). Immunoblots were performed on whole cell lysates with antibody to detect Beclin 1 and LC3II expression, or GAPDH for normalization of proteins. Representative immunoblots of Beclin 1 and LC3II proteins expression in *Salmonella*-infected Caco-2 cells in the presence of PD (**A**) or LY (**B**) are shown.

It was previously demonstrated [5] that the depletion of plasma membrane cholesterol suppressed Akt activation but enhanced ERK activation [5]. On the other hand, inhibition of PI3K/Akt signaling [18,19] and enhanced ERK1/2 activity [20,21] are involved in induction of autophagy, via regulating Beclin 1 [22]. To gain further insights into the involvement of Akt and ERK in the *Salmonella*-induced autophagy responses in IECs, Caco-2 cells were untreated or treated with PI3K inhibitor LY294002 or ERK inhibitor PD98059 and then infected by wild-type *S*. *typhimurium* strain SL1344. Western blot of Beclin 1 and LC3II was analyzed in whole cell lysates. As shown in Figure 2, neither LY294002 (LY) nor PD98059 (PD) had significant effect on Beclin 1 or LC3II expression in *Salmonella*-infected Caco-2 cells.

### 2.3. The Membrane Association of Nucleotide-Binding Oligomerization Domain-Containing Protein 2 (NOD2) and Atg16L1 Is Affected by the Depletion of Membrane Cholesterol

**Figure 3:**
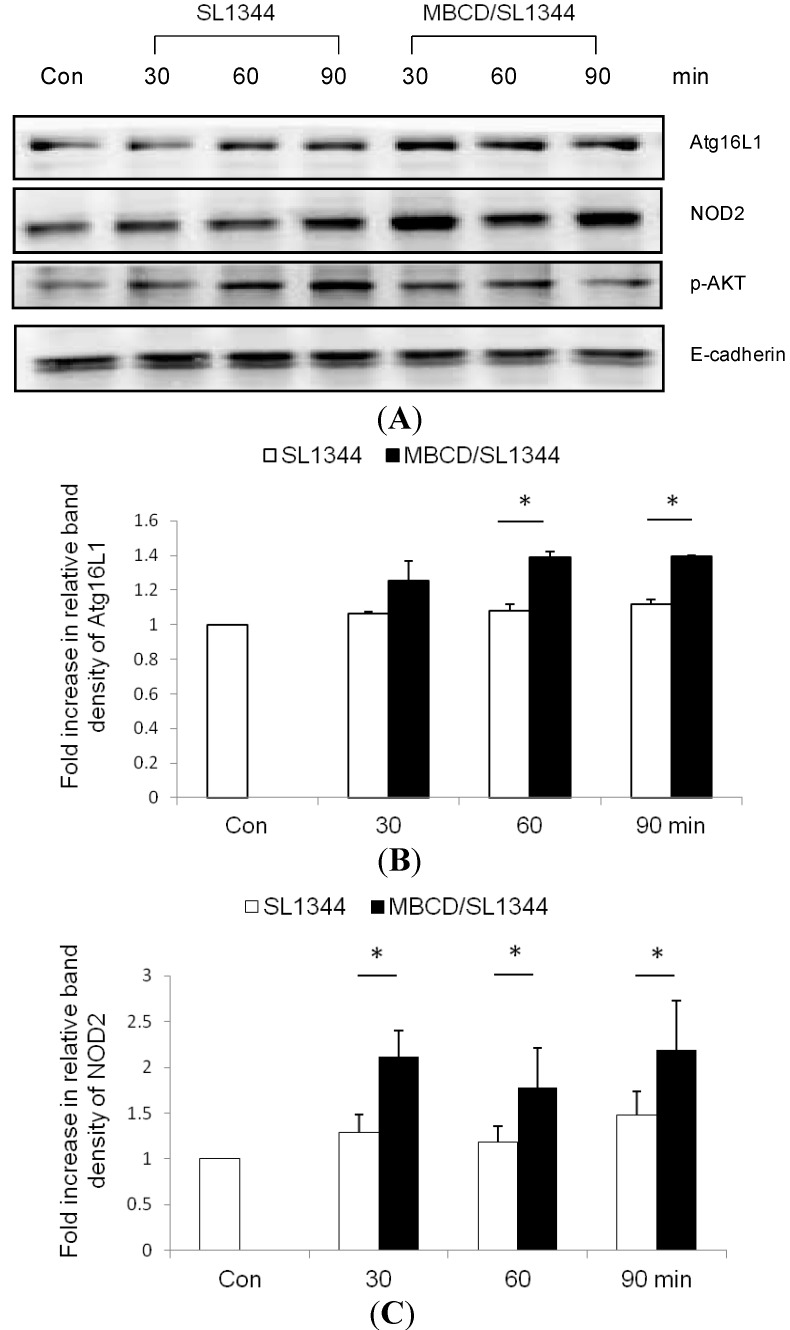
The effect of cholesterol on the membranous recruitment of nucleotide-binding oligomerization domain-containing protein 2 (NOD2) and Atg16L1 in *Salmonella*-infected Caco-2 cells. Caco-2 cells were uninfected (Con) or infected by wild-type *S*. *typhimurium* strain SL1344 for indicated times, in the presence or absence of MBCD. Immunoblots were performed on membrane lysates with antibody to detect NOD2, Atg16L1 and phosphorylated Akt proteins expression, and E-cadherin for normalization of membrane proteins. Representative immunoblots (**A**) and densitometric quantification of immunoreactive bands (**B**,**C**) are shown; The relative band intensities of Atg16L1 (**B**) and NOD2 (**C**) in untreated (white) and treated (black) Caco-2 cells were quantified as fold increases compared with the control cells. Each value represents the mean ± SEM of 3 independent experiments. An asterisk indicates a significant difference (*p* < 0.05).

The activated autophagy of epithelial cells, depending on nucleotide-binding oligomerization domain-containing protein 2 (NOD2) and Atg16L1 expression, increased the killing of *Salmonella* [15]. To examine whether or not depletion of membrane cholesterol affected the membrane recruitment of NOD2 and Atg16L1, subsequently leading to the activation of autophagy, Western blot of NOD2 and Atg16L1 proteins expression was analyzed in membrane extract of *Salmonella*-infected Caco-2 cells in the presence or absence of MBCD. As shown in Figure 3, depletion of membrane cholesterol by MBCD enhanced the NOD2 and Atg16L1 proteins expression in membrane fraction isolated from MBCD-treated *Salmonella*-infected Caco-2 cells.

### 2.4. Involvement of Atg16L1 in Enhancement of Salmonella-Induced Autophagy by Methyl-beta-cyclodextrin (MBCD)

**Figure 4:**
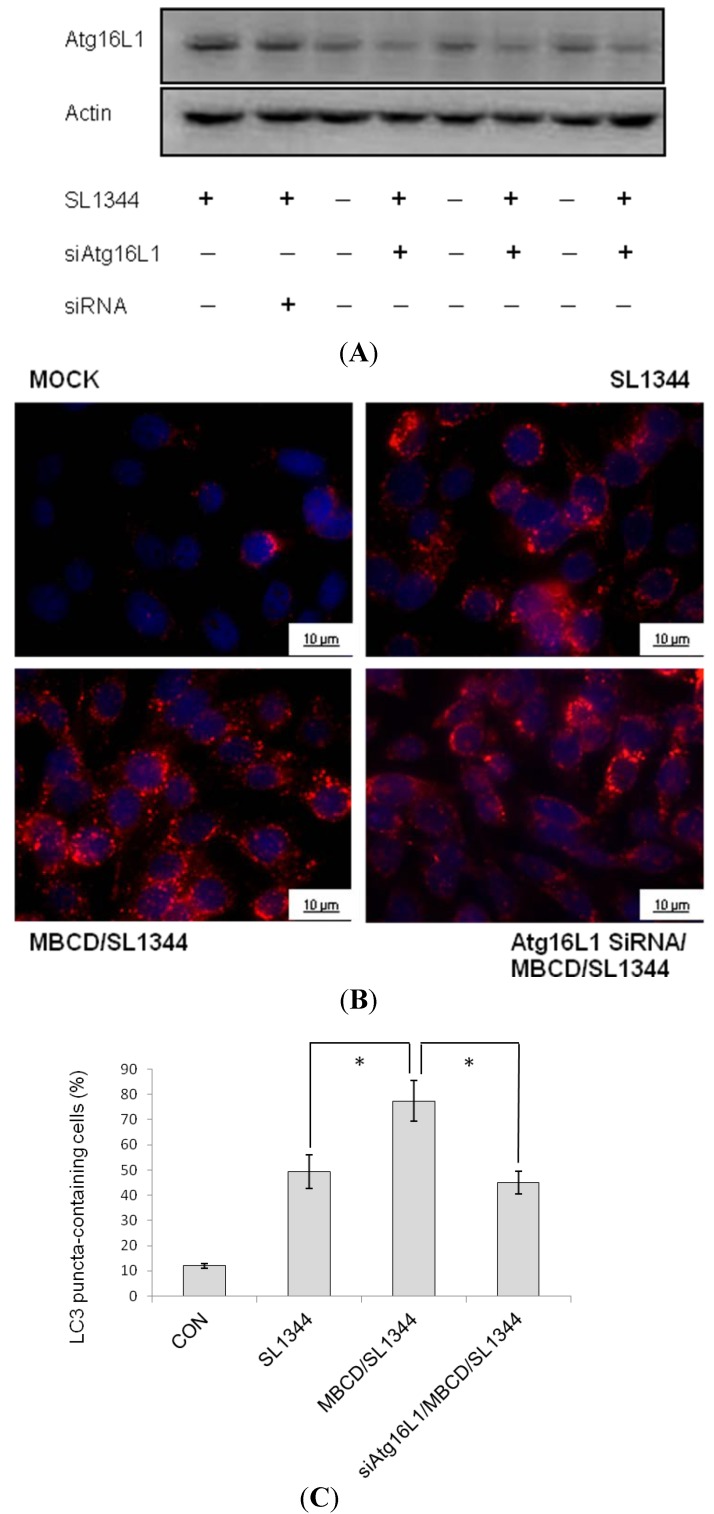
Involvement of Atg16L1 in enhanced *Salmonella*-induced autophagy by MBCD. Caco-2 cells were transfected with control siRNA or Atg16L1 siRNA (siRNA = non-target control siRNA; siAtg16L1 = siRNA to Atg16L1) for 48 h. The transfected cells were left uninfected (Con) or infected by wild-type *S*. *typhimurium* strain SL1344. (**A**) Western blots probed with antibodies against Atg16L1 and actin confirmed knockdown of Atg16L1. The transfected cells were pretreated with 4 mM MBCD for 30 min prior to wild-type *S*. *typhimurium* strain SL1344 infection for one hour. The cells were fixed, permeabilized and stained with anti-LC3 (red) and LC3 puncta formation was detected by a confocal microscope; (**B**) Representative images of LC3 punctae were depicted. Scale bar = 10 μm; and (**C**) The percentage of cells showing accumulation of LC3 punctae was reported. Data are mean ± SEM of three independent experiments. An asterisk indicates a significant difference (*p* < 0.05).

Finally, to examine the involvement of Atg16L1 in MBCD-mediated enhanced *Salmonella*-induced autophagy, Atg16L1 siRNA-transfected Caco-2 cells were untreated or pretreated by MBCD and then infected with wild-type *Salmonella* strain SL1344 for indicated times. Knockdown of Atg16L1 with specific siRNA in Caco-2 cells was confirmed by Western blot (Figure 4A). The Caco-2 cells were fixed, permeabilized and immunostained with antibody to endogenous LC3 and visualized by fluorescence microscopy. Immunofluorescence study (Figure 4B) showed numerous LC3 punctae (red) in *Salmonella*-infected cells. Pre-treatment of the cells with MBCD significantly increased the LC3 punctae while Atg16L1 siRNA-transfected Caco-2 cells significantly diminished MBCD-enhanced LC3 punctae to the level below SL1344 infection only. The MBCD-increased percentage of cells showing accumulation of LC3 punctae was significantly diminished by Atg16L1 siRNA (Figure 4C). Our results suggest that Atg16L1 becomes involved in the effect of MBCD on *Salmonella*-induced autophagy in Caco-2 cells.

### 2.5. Activation of NOD2 Suppressed the Activation of Akt in Salmonella-Infected Caco-2 Cells

**Figure 5:**
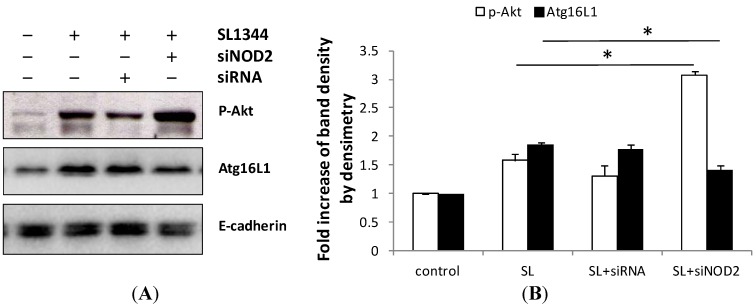
Effect of NOD2 knockdown on recruitment of Atg16L1 and activation of Akt in *Salmonella*-infected Caco-2 cells. Caco-2 cells were transfected with control siRNA (siRNA), and NOD2 siRNA (siNOD2) for 48 h and then uninfected or infected with wild-type *S*. *typhimurium* strain SL1344, as described in the Experimental Section. Proteins were harvested and Western blot analysis of phosphorylated Akt and Atg16L1 were done. E-cadherin was done for normalization of membrane proteins. Representative immunoblots (**A**) and densitometric quantification of immunoreactive bands of phosphorylated Akt (p-Akt) and Atg16L1 (**B**) are shown. The relative band intensities of p-Akt (white) and Atg16L1 (black) were quantified as fold increases compared with the control cells. Each value represents the mean ± SEM of 3 independent experiments. An asterisk indicates a significant difference (*p* < 0.05).

Muramyl dipeptide (MDP), a NOD2 ligand, significantly lowered the insulin-stimulated Akt phosphorylation. In order to investigate the effect of NOD2 on *Salmonella*-induced activation of Akt in IECs, Control siRNA or NOD2 siRNA-transfected Caco-2 cells were infected by SL1344. Knock-down of NOD2 was confirmed by Western blot with specific siRNA in Caco-2 cells up to 48 h in our previous study [23]. Western blot of phosphorylated Akt was analyzed in membrane extract of *Salmonella*-infected Caco-2 cells. As demonstrated in Figure 5, the *Salmonella*-induced phosphorylated Akt was enhanced in NOD2 siRNA-transfected Caco-2 cells. It suggests NOD2 negatively regulated the activation of Akt in *Salmonella*-infected Caco-2 cells.

To independently corroborate these findings, the results were confirmed in a different intestinal epithelial cell line, T84 cells, by using nystatin (a relatively mild agent that disassembles lipid rafts by binding cholesterol), which did not affect cell viability (data not shown). It was demonstrated that disruption of membrane cholesterol by nystatin enhanced NOD2 and Atg16L1 proteins expression in membrane, and autophagic LC3II protein expression in *Salmonella*-infected T84 cells, in supplemental Figure 1.

### 2.6. Discussion

Cholesterol depletion was found to activate macroautophagy in several cell types [24]. In Figure 1, it was demonstrated disruption of membrane cholesterol by methyl-beta-cyclodextrin (MBCD) enhanced the LC3II expression, a central autophagy marker, in *Salmonella*-infected Caco-2 cells. Epithelial cell MyD88 signaling is required for *Salmonella* induction of autophagy in IECs [25] and MyD88 could target Beclin 1 to trigger autophagy in macrophages [26]. So it was hypothesized that MBCD might enhance autophagy via enhancement of Beclin 1 expression. However, MBCD did not have a significant effect on Beclin 1 expression (Figure 1). It suggests that the enhancement of autophagy by MBCD is Beclin 1-independent. Recently, non-canonical, Beclin 1-independent autophagy has been reported [27] and the phagophore precursor is formed directly from existing plasma membrane. However, the interaction of MyD88 with Beclin 1 may reduce the binding of Beclin 1 to Bcl-2, leading to the apoptosis of the vacuole (damaged vacuole). In addition, MyD88-mediated autophagy [26,28,29] will fuse with lysosome for degradation of the damaged SCV.

As demonstrated in the previous study, MBCD suppressed the activation of Akt (Figure 3). Nevertheless, neither PI3K inhibitor LY294002 nor ERK inhibitor PD98059 had significant effects on LC3II autophagy protein expression in *Salmonella*-infected Caco-2 cells (Figure 2). It suggested ERK and PI3K/Akt signals play selective roles on inflammation and apoptosis [6] but not on autophagy in *Salmonella*-infected Caco-2 cells, though plasma membrane cholesterol plays a critical role on the *Salmonella*-induced PI3K/Akt and ERK signals [5].

The plasma membrane contributes directly to the formation of Atg16L1-positive early autophagosome precursors [2], which subsequently mature to form autophagosome. NOD2 localization at the membrane directs autophagy by recruiting Atg16L1 to the plasma membrane [11] at the bacterial entry site of intestinal epithelial cells [15]. Atg16L1 is required for autophagy in IECs and protection of mice from *Salmonella* infection [30]. Taken together, this explains the finding that plasma membrane cholesterol depletion by MBCD enhanced *Salmonella*-induced autophagy (Figure 4) via enhanced membrane NOD2 and Atg16L1 proteins expression (Figure 3). Depletion of cholesterol in *Mycobacterium avium*-infected macrophages [31] triggers phagolysosomal fusion and autophagic degradation of the bacteria. Therefore, cholesterol accumulation in pathogenic vacuoles could provide the vacuole-bound pathogens a strategy to invade and establish a replicative vacuole [32]. Autophagy triggered by depletion of cholesterol can recognize intracellular *Salmonella enterica* serovar Typhimurium in damaged vacuoles [7] or the *Salmonella* released into cytosol, bringing about the clearance of the pathogen [33], and protection against *S. typhimurium* infection [34]. Therefore, disruption of cellular cholesterol may result in damaged SCV [32] which is degraded by enhanced Atg16L1-mediated autophagy. This is the first time to demonstrate that accumulated membrane cholesterol at the bacterial entry site selectively co-opts autophagy complexes through different proteins to subvert host clearance and promote intracellular bacterial infection. Clinically, gastrointestinal infections with pathogens like *Salmonella* can trigger or increase the likelihood that a patient will subsequently develop inflammatory bowel disease [35]. Decreased intracellular clearance of *Salmonella* in cells having the Crohn’s disease (CD)-associated Atg16L1 variant [14,16,36]. It is proposed that the association of ATG16L1 mutant with increased risk of CD is due to impaired handling of intracellular bacteria by autophagy [13]. The drugs targeting membrane cholesterol may induce autophagic clearance of invading pathogens and lower the risk of Crohn’s disease.

It is well known that Akt plays an anti-apoptotic role on maintenance of SCV in IECs [37]. It was observed NOD2 negatively regulated the activation of Akt (Figure 5), may lead to apoptosis of infected SCV [38] and release of *Salmonella* into cytosol, which is degraded by the Atg16L1-mediated autophagy [30].

## 3. Experimental Section

### 3.1. Reagents

The ERK inhibitor PD98059 and PI3K inhibitor LY294002, were obtained from Calbiochem (San Diego, CA, USA) and stock solutions made in dimethylsulfoxide (DMSO, Sigma-Aldrich, St. Louis, MO, USA). The inhibitor was added to cells at the specified concentrations about 60 min before infection. Cholesterol-extractor (methyl-beta-cyclodextrin) or cholesterol-binding (nystatin) agents were purchased from Sigma-Aldrich Co. (St. Louis, MO, USA). Standard laboratory reagents were from Sigma-Aldrich (St. Louis, MO, USA) or Fisher Scientific (Pittsburgh, PA, USA).

### 3.2. Bacterial Strains

The wild-type *Salmonella* enterica serovar *typhimurium* (*S. typhimurium*) strain used in this work was SL1344 which has been described previously [5,6,39,40]. All bacteria were grown overnight in static cultures with minimal aeration in Luria-Bertani (LB) medium. The bacteria were collected by centrifugation at 14,000× *g* for 5 min, washed with sterile phosphate-buffered saline (PBS), and resuspended in tissue culture medium without antibiotics at a density of 4 × 10^9^/mL. Aliquots of this suspension (25 or 50 μL; (1–2) × 10^8^ bacteria) were used to infect the cells. The bacterial inoculum was adjusted to a bacteria to cell ratio of 200:1.

### 3.3. Cell Culture and Infection

Caco-2 or T84 cells (ATCC, Rockville, MD, USA) were grown as previously reported [5,6,39,40] to 50%–75% confluence. About 30 min before addition of bacteria, the cells were washed and placed in antibiotic-free medium, and pharmacological inhibitors added at this time in some experiments.

### 3.4. Depletion of Cellular Cholesterol

To remove cholesterol, serum-starved cells were incubated in DMEM ( Sigma-Aldrich, St. Louis, MO, USA) containing the indicated concentration of methyl-beta-cyclodextrin (MBCD, Sigma-Aldrich, St. Louis, MO, USA) or Nystatin (Sigma-Aldrich, St. Louis, MO, USA) for 30 min at 37 °C, as in our previous report [5]. The efficiency of cholesterol extraction has been reported in previous studies [41]. The efficiency of cholesterol depletion has been confirmed by measuring the fraction of free cholesterol remaining after treatment and observed MBCD or Nystatin (Sigma-Aldrich, St. Louis, MO, USA) by itself did not induce autophagy protein LC3II expression in Caco-2 or T84 cells (data not shown).

### 3.5. Cell Fractionation

Cytosol and membrane fractions were prepared as described [5,42]. Briefly, cells were suspended in hypotonic lysis buffer (25 mM Tris, pH 7.4, 5 mM EDTA, 1 mM dithiothreitol, 1 mM Na_3_VO_4_, 2.5 mM Na_4_P_2_O_7_, and protease inhibitors) and disrupted by five passages through a 27-gauge needle (Thomas Scientific, Swedesboro, NJ, USA). Large cell debris was pelleted by centrifugation at 2000× *g* for 5 min at 4 °C. The supernatants were centrifuged at 100,000× *g* for 20 min at 4 °C. The resulting supernatant (cytosol) was collected, and the pellet was resuspended in lysis buffer containing 1% Triton X-100 (Sigma-Aldrich, St. Louis, MO, USA). The lysate was again centrifuged at 100,000× *g* for 20 min at 4 °C, and the supernatant (membrane) was collected. The distributions of proteins in the cytosol and membrane fractions were analyzed by Western blotting.

### 3.6. Western Blot

Equal amounts of total protein were separated by SDS-PAGE and then transferred to nitrocellulose membranes (Thermo Fisher Scientific Inc., Rockford, IL, USA) by semi-dry blotting as previously described [5,6,39,40]. After blocking the membranes with 5% non-fat dry milk, they were probed with antibodies to either phosphorylated Akt (Cell Signaling Technology, Danvers, MA, USA), phosphorylated ERK (Santa Cruz Biotechnology, Dallas, Texas, TX, USA), or LC3-II, Beclin 1 (Cell Signaling Technology, Danvers, MA, USA), or NOD2, Atg16L1 (Labome Org, Princeton, NJ, USA), and then developed with horseradish peroxidase-conjugated second antibodies (Zymed Laboratories, San Francisco, CA, USA) and enhanced chemiluminescence (Pierce Chemical Co., Rockford, IL, USA). Appropriate exposures to X-ray film were made, and the filters were then stripped and re-probed with antibodies to E-cadherin or GAPDH (Santa Cruz Biotechnology, Dallas, Texas, TX, USA) as appropriate.

### 3.7. Real-Time PCR for mRNA Assay

Total RNA was prepared from control or infected cells with the Trizol reagent (Invitrogen Corporation, Carlsbad, CA, USA), following the manufacturer’s directions. The RNA was reverse transcribed with random hexamers using the GeneAmp kit (Roche, Nutley, NJ, USA) as described in detail earlier [5,6,23,40]. Real-time reverse transcription-PCR analyses were performed in a fluorescence temperature cycler (LightCycler; Roche Diagnostics, Indianapolis, IN, USA) as described previously [5,23]. The following primers were used: NOD2, 5'-AGCCATTGTCAGGAGGCTC-3' (forward primer) and 5'-CGTCTCTGCTCCATCATAGG-3' (reverse primer); Atg16L1, 5'-ACGTACCAAACAGGCACGAG-3' (forward primer) and 5'-CAGGTCAGAGATAGTCTGCAAAC-3' (reverse primer); glyceraldehydes-3-phosphate dehydrogenase, 5'-CCAGCCGAGCCACATCGCTC-3' (forward primer) and 5'-ATGAGCCCCAGCCTTCTCCAT-3'. All quantifications were normalized to the housekeeping gene glyceraldehyde-3-phosphate dehydrogenase. Relative expression was given as a ratio between target gene expression and glyceraldehyde-3-phosphate dehydrogenase expression.

### 3.8. Small-Interfering RNA (siRNA) Transfection

All transient transfections were carried out in triplicate using *NeoFX* reagent (Ambion, Austin, TX, USA) to final concentration of 20 nM following the manufacturer’s instructions. RNAi experiments in Caco-2 cells were done as described previously [23], including control nonsilencing small interference RNA (siRNA), NOD2siRNA and different siRNAs targeting Atg16L1 (sequence 1: sense GAGUUGUCUUCAGCCCUGAUGGCAG, antisense CUGCCAUCAGGGCUGAAGACAACUC; sequence 2: sense GGCUCUGCUGAGGGCUCUCUGUAUA, antisense UAUACAGAGAGCCCUCAGCAGAGCC; sequence 3: sense CAAGGGUUCCCUAUCUGGCAGUAAU, antisense AUUACUGCCAGAUAGGGAACCCUUG (sequence were purchased from Invitrogen Corporation, Carlsbad, CA, USA). All siRNA were tested and verified as reducing expression by >80% protein reduction in Caco-2 cells by immunoblot analysis or reducing expression of >50% of mRNA by real-time PCR when appropriate Ab was not available. For the Caco-2 cells, 20nM of each siRNA was transfected 48–96 h before *S. typhimurium* infection.

### 3.9. Immunofluorescence Analysis

After infection and treatment, Caco-2 cells in culture were washed three times with ice-cold PBS, fixed in 4% paraformaldehyde for 15 min, treated for 15 min in 50 mM NH_4_Cl, then blocked and permeabilized for 30 min in 1% bovine serum albumin (BSA, Thermo Fisher Scientific Inc., Rockford, IL, USA)–0.2% Triton X-100. Cells were incubated with rabbit anti-LC3B (Cell Signaling Technology, Danvers, MA, USA) at 1:250 dilution in blocking buffer for 1 h. Secondary antibody was goat anti-rabbit IgG conjugated with Alexa Fluor 594 fluorochrome (Invitrogen Molecular Probes, Eugene, OR, USA). The nuclear morphology was visualized with the fluorescent dye Hoechst (Sigma Aldrich, St. Louis, MO, USA). Then the coverslips were mounted on slides with Vectashield (Vector Labs, Burlingame, CA, USA) mounting medium, which were examined under immunoflourescence conditions by using a Zeiss Axio Observer Z1 (Carl Zeiss, Jena, Germany). The percentage of cells with endogenous LC3 punctae was determined by counting the number of positively stained cells from 100 randomly chosen cells from three separate experiments.

### 3.10. Cell Viability and Morphologic Features

Representative cell populations from each condition were examined under light microscopy. No significantly morphologic change was observed under any condition. Cell viability was also confirmed by trypsan blue exclusion.

### 3.11. Statistical Analysis

All experiments were carried out at least twice with similar results. Statistical significance was determined using the student’s *t* test. Comparisons with a *p* value of <0.05 were considered statistically significant.

## 4. Conclusions

In conclusion, disruption of membrane cholesterol by MBCD recruited NOD2 into membrane, in one way, to suppress the PI3K/Akt-mediated anti-apoptotic responses, resulting in damaged SCV; and in another way, to enhance Atg16L1 expression, leading to autophagic clearance of the damaged SCV. It suggests that cholesterol accumulation in plasma membrane at the entry site of *Salmonella* activated PI3K/Akt signaling, bringing about the formation of SCV but suppressed membrane recruitment and expression of NOD2 and Atg16L1, giving rise to decreased autophagy. This study provides an opportunity to understand the mechanism of *Salmonella*-induced innate immunity in IECs, which in turn may lead to development of new therapeutic approaches not only for Crohn’s disease, but also for intestinal infection and inflammation in general.

## Supplementary Files

Supplementary File 1
