# Supplementary material for: The Critical Role of Membrane Cholesterol in Salmonella-Induced Autophagy in Intestinal Epithelial Cells
Source: Int J Mol Sci. 2014 Jul 15;15(7):12558–72. doi: 10.3390/ijms150712558 (PMC4139860; doi:10.3390/ijms150712558)
Supplement: Supplementary File 1 [file ijms-15-12558-s001.doc]

**Supplementary Information**

**Figure S1.** The role of cholesterol on the autophagy proteins expression in *Salmonella*-infected T84 cells. T84 cells were untreated (0 min) or treated with nystatin and then infected by *Salmonella typhimurium* wild-type strain SL1344 for indicated time. Immunoblots were performed on cytosolic and membrane lysates with antibody to detect LC3B, phosphorylated Akt, NOD2 and Atg16L1 expression, or actin and E-cadherin for normalization of cytosolic and membrane proteins, respectively. Representative immunoblots are shown.
